# Supplementary material for: Whole-Genome Analysis of Porcine Circovirus Type 2 in Russia
Source: Pathogens. 2021 Dec 16;10(12):1631. doi: 10.3390/pathogens10121631 (PMC8705920; doi:10.3390/pathogens10121631)
Supplement: Supplementary file 1 [file pathogens-10-01631-s001.zip › pathogens-1447435-supplementary.pdf]

Table S1. Supplementary. PCV2 strains from Russia used in this study.

№	PCV2 strain	Year of isolation	Region of Russia	Genotype	Genome, nt	ORF2 size, nt	GenBank accession number	Health status	Age	Sample	Number of samples used
1	Belgorod_M_18	2018	Belgorod	2d	1767	705	MZ511702	PDNS	fattening pigs	tissue homogenates	13
2	Belgorod_RA18	2018	Belgorod	2a	1757	702	MZ511700	Non-reported	fattening pigs	tissue homogenates	20
3	Burytia2018	2018	Burytia	2d	1767	705	MZ511694	Non-reported	growing pigs	tissue homogenates	2
4	Krasnoyarskiy2018	2018	Krasnoyarskiy	2d	1767	705	MZ511703	PDNS	fattening pigs	tissue homogenates	10
5	Tomskaya18	2018	Tomskaya	2d	1767	705	MZ511698	Non-reported	fattening pigs	tissue homogenates	4
6	Smolenskaya2018	2018	Smolenskaya	2d	1767	705	MZ511697	Non-reported	growing pigs	tissue homogenates	6
7	Sverdlovskaya18	2018	Sverdlovskaya	2d	1767	705	MZ511696	PDNS	fattening pigs	tissue homogenates	4
8	Vologodskaya2018	2018	Vologodskaya	2b	1767	702	MZ511695	Non-reported	suckling pigs	tissue homogenates	5
9	Kemerovo_Ch_522	2019	Kemerovo	2d	1767	705	MZ511693	PDNS	growing pigs	serum	8
10	Kemerovo_Sl_809	2019	Kemerovo	2d	1767	705	MZ511691	PDNS	fattening pigs	serum	30
	Kemerovo_Sl_1405	2019	Kemerovo	2d	1767	705	MZ511692	PDNS	fattening pigs	serum	30
12	Moskovskaya_2020	2020	Moskovskaya	2d	1767	705	MZ511701	Non-reported	growing pigs	serum	13
13	Ryazanskaya_2020	2020	Ryazanskaya	2d	1767	705	MZ511699	Non-reported	fattening pigs	tissue homogenates	5
14	Kurskaya_2020	2020	Kurskaya	2d	1767	705	MZ511704	Non-reported	fattening pigs	tissue homogenates	5
